# Suitability of raw and heat-treated *Amaranthus spinosus* in broiler diets: Effects on growth performance, meat antioxidant capacity, haemato-biochemical parameters, intestinal histomorphometry, and cecal volatile fatty acid profile

**DOI:** 10.1007/s11250-024-04099-4

**Published:** 2024-08-10

**Authors:** Emre Sunay Gebeş, Sakine Yalçın, Muhammad Shazaib Ramay, Akın Ünal, Kürşat Filikçi

**Affiliations:** 1 Faculty of Veterinary Medicine, Dept. of Animal Nutrition and Nutritional Diseases, Ankara University, Ankara, Türkiye; 2 Faculty of Veterinary Medicine, Dept. of Microbiology, Ankara University, Ankara, Türkiye; 3 Faculty of Veterinary Medicine, Dept. of Pathology, Harran University, Ankara, Türkiye

**Keywords:** *Amaranthus spinosus*, Broiler performance, Antioxidant potential, Intestinal histomorphometry, Haemato-biochemical characteristics

## Abstract

This study aimed to examine the effects of incorporating amaranth (*Amaranthus spinosus*, either raw or heat-treated) into broiler diets on growth performance, meat antioxidant capacity, haemato-biochemical parameters, intestinal histomorphometry, and cecal volatile fatty acid profile. A total of 210 male Ross 308 broiler chicks were allocated to five dietary treatments in a completely randomized design, with each treatment comprising six replicates of seven birds each. The control group received a diet based on maize and soybean meal, while the remaining dietary groups were formulated to be isonitrogenous and isocaloric to the control, with exact levels of 10% and 20% raw or heat-treated amaranth in the diet. Body weight and feed intake were monitored on days 0, 10, 24, and 39 of the study. On day 39, two birds per replicate were randomly selected for blood sampling, followed by slaughtering for further parameter examination. Incorporating A. spinosus up to 20% in broiler diets had no adverse effect on body weight gain compared to the control. However, higher levels of amaranth led to a negative impact on the feed conversion ratio, attributed to increased feed intake. Furthermore, amaranth supplementation did not negatively influence carcass yield or various organ weights, except for the gizzard, which was heavier in the amaranth-fed groups. Notably, amaranth supplementation reduced abdominal fat, enhanced meat antioxidant status, and had no detrimental effects on blood biochemical or hematological indices. Additionally, amaranth feeding resulted in decreased blood triglyceride levels but had no effect on cholesterol levels. While heat treatment of amaranth did not significantly alter the performance of broiler chickens, it enhanced the beneficial effects of amaranth feeding on the histomorphological features of the duodenum and ileum, and increased blood IgG levels. The cecal volatile fatty acid profile remained largely unaffected by amaranth inclusion, although heat-treated amaranth led to increased levels of branched-chain fatty acids and valerate. Overall, the findings suggest *A. spinosus* as a promising alternative feed ingredient for broilers when included at 10% of the diet. However, further research is needed to investigate the effect of various amaranth species, processing methods and enzyme supplementation on poultry nutrition to expand its inclusion rate.

## Introduction

Cereals, legume seeds and their byproducts make significant contributions as raw feed materials to all livestock enterprises, particularly in poultry production. Not only are they important sources of energy and protein, but they also provide compounds with biological activity. However, the increasing demand for animal protein, combined with a scarcity of livestock feed resources and associated high feed costs, necessitates the investigation of alternatives that can provide at least the same nutrition as conventional feed materials. Natural remedies for sustainable agriculture have therefore brought renewed interest to useful but often neglected crops (Borelli et al. 2020).

One such crop is amaranth (*Amaranthus spp.*), a pseudocereal widely grown in the tropics that has promising economic value due to its ability to withstand a wide range of climatic and agronomic conditions as well as its unique nutritional, functional and technological capacities (Manyelo et al. 2020). The Amaranth genus includes approximately 70 species of annual plants. Some of these species, such as *A. cruentus*, *A. hypochondriacus,* and *A. caudatus*, were domesticated primarily for edible grain, while others, including *A. dubius, A. blitum and A. tricolor*, were cultivated for green vegetables (Das 2012; Aderibigbe et al. 2022). Additionally, some species are currently considered weeds until their potential is fully realized and more suitable uses are discovered. From a nutritional standpoint, grain amaranth is a good source of lipids (linoleic and oleic acids), quality proteins (largely albumins with balanced amino acid composition, including lysine and sulfur-containing amino acids), minerals (calcium, phosphorus, potassium, magnesium, iron) and vitamins (B-group vitamins, and vitamins A, E, and C), especially when compared to conventional cereals. Moreover, amaranth plants are unique in terms of their health-beneficial phytochemicals, which include various phytosterols and tocopherols, as well as high levels of squalene (Gamel et al. 2006; Baraniak et al. 2022).

The literature indicates that raw amaranth seeds do not naturally exhibit the protein quality suggested by their amino acid composition. The presence of various antinutrients in amaranth reduces the availability of essential amino acids, and interferes with the metabolic processes of other nutrients (Manyelo et al. 2020). This is one of the primary reasons why livestock producers reject unconventional feed materials. To mitigate antinutrient effects and successfully incorporate amaranth into animal feeding programs while adhering to health recommendations, a variety of techniques have been proposed. Due to the heat-lability of most antinutrients, thermal processing techniques (roasting, flaking, popping, wet cooking, autoclaving, or extruding) are most effective at improving the nutritional value of feeds. In a study where broilers were fed grain amaranth, heat treatment improved the metabolizable energy content of amaranth by 573 kcal/kg compared to the untreated amaranth (Janmohammadi et al. 2022). The authors observed that heat-treated amaranth exhibited up to twice the crude protein content and a comparable available metabolizable energy value to that of corn grain. This makes amaranth an advantageous feed material with the potential to uphold performance targets in tandem with health, welfare, and economic aspects of animal production.

In recent years, concerted efforts have been made to explore the incorporation of grain amaranth into poultry diets as a strategy to replace traditional feed resources. A review of the literature revealed maintenance or improvement in performance indices (Popiela et al. 2013; Hosseintabar-Ghasemabad et al. 2022), hypocholesterolemic effects (Alizadeh-Ghamsari et al. 2021), improved antioxidant status and atherogenic indices (Hosseintabar-Ghasemabad et al. 2022), improved animal health and production of healthier products (Popiela et al. 2013; Janmohammadi et al. 2023) when amaranth was incorporated into poultry diets, either raw or processed, or with other additives. Furthermore, when different amaranth species were evaluated, records revealed significant variability in terms of essential nutrients (Szabóová et al. 2020), phytochemical and antinutrient levels (Mlakar et al. 2009). Research has also indicated significant variability in poultry production responses, attributed to differences in both thermal processing methods and amaranth species (Kianfar et al. 2023; Hosseintabar-Ghasemabad et al. 2024). Thus, when formulating animal diets with amaranth grains, plant species- and processing-related differences in feed value should also be considered.

To date, animal studies have focused primarily on grain-type amaranth, specifically *A. cruentus* and *A. hypochondriacus*. There is hardly any information on the use of weed amaranths in poultry diets. *A. spinosus*, commonly known as spiny amaranth or pigweed, was found to exhibit significant immunomodulatory, anti-inflammatory, antimicrobial, and antioxidant activities (Adegbola et al. 2020). Therefore, the present study aimed to examine the effects of incorporating of *A. spinosus* (either raw or heat-treated) into broiler diets on growth performance, meat antioxidant capacity, haemato-biochemical parameters, intestinal histomorphometry, and cecal volatile fatty acid profile.

## Materials and methods

All animal care and use procedures were approved by the Animal Ethics Committee of Ankara University (2017–26-209).

### Procurement and heat treatment of amaranth (*A. spinosus*) grains

The amaranth seeds were procured from a local distributor, Yayla Agro Inc., which imported them from India. After species identification and confirmation with the Landscape Architecture Department, Faculty of Agriculture, Ankara University, the seeds were heat-treated to reduce the negative effects of antinutrients. A twin-screw extruder (DG-85, Jinan, China) without a die was used to achieve controlled and uniform cooking. Following a few exploratory first experiments, the barrel temperature and seed flow rate were standardized to produce a final product with a temperature of 70–75°C. The seeds were subsequently introduced to a heated barrel with a temperature profile of 106°C, 122°C, and 180°C during treatment. The device was run at the slowest possible speed (20 Hz / inverter driven), and it took approximately 25–30s for the seeds to travel from the entrance to the exit point. Heat-treated amaranth seeds were instantly spread out and cooled by convection before being properly packed. No popping was observed in the seeds, but they did have a slightly roasted color.

### Birds, diets, and experimental design

A total of 210, day-old male Ross 308, broiler chicks were obtained from a commercial hatchery (BeyPi Inc., Ankara, Türkiye) and were randomly allocated to five experimental groups of six replicates each and 7 birds/replicate. Chicks were spray-vaccinated in the hatchery before arrival. The control group was fed a standard mix of maize and soybean meal (Table 1), which was prepared following Aviagen (2019) recommendations. The four other diets were obtained by substituting raw (**RW**) and heat-treated (**HT**) amaranth for portions of corn and soybean up to 10 and 20% of the diet (RW_10_, RW_20_, HT_10_, and HT_20_). All the diets were isonitrogenous and isocaloric. Proximate analysis of amaranth seeds and experimental diets was performed according to the methods described in AOAC (2000).

**Table 1:** Ingredients and composition of experimental diets (as-fed basis)

	0-10d					11-24d					25-39d				
		Treatments^1^													
Ingredient, %	C	RW_10_	RW_20_	HT_10_	HT_20_	C	RW_10_	RW_20_	HT_10_	HT_20_	C	RW_10_	RW_20_	HT_10_	HT_20_
Corn	46.50	39.50	32.59	39.50	32.59	49.70	42.58	35.47	42.58	35.47	49.91	42.87	35.83	42.87	35.83
Soybean mea	22.39	21.39	21.00	21.39	21.00	18.50	18.00	17.50	18.00	17.50	20.23	19.72	19.21	19.72	19.21
Full-fat Soya	25.00	23.00	20.30	23.00	20.30	26.25	23.87	21.48	23.87	21.48	22.41	19.96	17.51	19.96	17.51
Soybean Oil	1.50	1.50	1.50	1.50	1.50	2.00	2.00	200	2.00	2.00	4.10	4.10	4.10	4.10	4.10
Amaranth	-	10.00	20.00	10.00	20.00	-	10.00	20.00	10.00	20.00	-	10.00	20.00	10.00	
Limestone	1.00	1.00	1.00	1.00	1.00	1.00	1.00	1.00	1.00	1.00	1.00	1.00	1.00	1.00	1.00
DCP	2.30	2.30	2.30	2.30	2.30	1.30	1.30	1.30	1.30	1.30	1.20	1.20	1.20	1.20	1.20
Methionine	0.40	0.40	0.40	0.40	0.40	0.40	0.40	0.40	0.40	0.40	0.35	0.35	0.35	0.35	0.35
Lysine	0.20	0.20	0.20	0.20	0.20	0.20	0.20	0.20	0.20	0.20	0.15	0.15	0.15	0.15	0.15
Threonine	0.10	0.10	0.10	0.10	0.10	0.10	0.10	0.10	0.10	0.10	0.10	0.10	0.10	0.10	0.10
Salt	0.30	0.30	0.30	0.30	0.30	0.30	0.30	0.30	0.30	0.30	0.30	0.30	0.30	0.30	0.30
Vit-Premix^2^	0.15	0.15	0.15	0.15	0.15	0.15	0.15	0.15	0.15	0.15	0.15	0.15	0.15	0.15	0.15
Min-Premix^3^	0.10	0.10	0.10	0.10	0.10	0.10	0.10	0.10	0.10	0.10	0.10	0.10	0.10	0.10	0.10
Anticoccidial^4^	0.06	0.06	0.06	0.06	0.06	-	-	-	-	-	-	-	-	-	-
Chemical composition															
ME (kcal/kg)	3020	3019	3012	3000	3014	3114	3096	3092	3095	3097	3219	3225	3231	3234	3228
Crude Protein (%)	22.97	22.79	22.64	22.77	22.65	21.82	21.73	21.65	21.72	21.62	21.05	21.03	21.03	21.10	21.01
Crude Fiber (%)	3.00	3.16	3.36	3.14	3.30	2.83	3.02	3.23	3.00	3.25	2.80	3.00	3.10	2.98	3.17

^1^C, control, non-supplemented diet; RW_10_ and RW_20_, raw amaranth incorporated groups at levels 10 and 20% of the diet, respectively; HT_10_ and HT_20_*,* heat-treated amaranth-incorporated groups at levels 10 and 20% of the diet, respectively

^2^Vitamin premix (1 kg) 11.000.000 IU vitamin A, 3.500.000 vitamin D3, 100 g vitamin E, 3 g vitamin K3, 3 g Vitamin B1, 6 g Vitamin B2, 15 g calcium, D-pantothenate, 1 g vitamin B6, 20 mg vitamin B12, 35 g niacin, 1.5 g folic acid, 200 mg biotin

^3^Mineral premix (1 kg) 30 g copper, 120 g manganese, 110 g zinc, 2 g iodine, 300 mg selenium, 50 g iron

^4^Coxidin®

In all feeding phases (starter, 0–10 days; grower, 11–24 days; and finisher, 25–39 days), the birds were given ad libitum access to feed (mash form) and water. Each pen was equipped with a plastic feeder and automatic nipple drinkers. The birds were reared in an environmentally controlled house, starting at an initial temperature of 33°C, which was gradually decreased to 22°C over a 3-week period and then maintained thermostatically. Each pen (90 cm length × 80 cm width × 80 cm height) had wood shavings as litter. The relative humidity of the house during the experiment was 50 ± 5%. The house was artificially ventilated, and the lighting schedule was 24L:0D for the first three days, reduced further to 23L:1D from d 4–7, and then kept constant at 20L:4D thereafter.

### Data collection and sampling

On days 0, 10, 24 and 39 of the experiment, all the birds were individually weighed, and feed intake (**FI**) was recorded. Mortalities were monitored daily. Body weight gain (**BWG**) and the feed conversion ratio (**FCR**) were subsequently calculated to evaluate growth performance. The FCR was calculated as the ratio of FI to BWG (g/g). On day 39, based on the group average weight, two birds from each replicate were randomly selected. A total of 12 individual blood samples per group (unfasted) were collected by a veterinarian using vacuum tubes with (**EDTA**) and without anticoagulant. Approximately 3 ml of blood/tube was drawn from the brachial vein of each bird. Following blood collection, the birds were slaughtered and properly eviscerated. Hot carcass weights were then recorded, and carcass yield (relative to pre-slaughter live weight) was determined. The weights of various organs (liver, heart, empty gizzard, spleen, and bursa Fabricius) and abdominal fat were recorded and expressed relative to the pre-slaughter live weight. The pH of the fresh breast meat samples was measured within 15 min after slaughter using a pH meter (Mettler Toledo, SevenGoTM, pH meter SG2, puncture pH electrode LE427).

### Breast meat antioxidant status

Following carcass evaluation, breast meat samples (pectoralis major) were collected and stored at ­20ºC before analysis. The antioxidant status of the broiler breast meat was evaluated in terms of 2,2-diphenyl-1-picrylhydrazyl (**DPPH**) radical scavenging activity, total antioxidant status (**TAS**), total oxidant status (**TOS**), and oxidative stress index (**OSI**) (Ramay et al. 2020). Furthermore, catalase (**CAT**) and myeloperoxidase (**MPO**) enzyme activities were measured using commercially available kits (Rel Assay Diagnostic Kits) according to the manufacturer’s protocol.

### Analyses of blood samples

After the blood was collected, it was allowed to rest for 15 min and then centrifuged (1,690 × g for 10 min at 4°C; SL 16R centrifuge, Thermo Scientific, Osterode am Harz, Germany) to obtain serum. TAS, TOS, and OSI analyses were performed on the blood serum as described elsewhere (Ramay et al. 2020). Blood serum samples were also analyzed for total protein (**TP**), albumin (**ALB**), cholesterol (**CHL**), and triglyceride (**TG**) levels with an autoanalyzer (BT 3000, Biotechnica Instruments) using commercial kits from the Randox RX series (Randox Laboratories). The immunoglobulin IgG concentration was analyzed using an ELISA kit (Randox, IG3896) according to the manufacturer’s protocol. The hematological parameters (red blood cells, **RBCs**; hematocrit, **HTC**; hemoglobin, **Hb**; white blood cells, **WBC**; platelets, **PLT**; mean corpuscular hemoglobin, **MCH**; mean corpuscular hemoglobin concentration, **MCHC**; mean corpuscular volume, **MCV**; red cell distribution width, **RDW**) were evaluated within two hours after blood sampling (EDTA) using an automated hematology analyzer (Sysmex pocH-100iV, Sysmex Corporation, Japan).

### Histomorphological measurements of the small intestine

Alter slaughtering, intestinal tract was removed immediately. For the sample uniformity, approximately 2 cm lengths of mucosal segments of duodenum, jejenum and ileum were excised as follows: duodenum (from gizzard outlet to the end of the pancreatic loop), jejenum (8 cm proximal to Meckel’s diverticulum), and ileum (8 cm proximal to the ileo-cecal junction). Samples for histomorphological analysis were taken from the duodenum, jejunum and ileum. The tissue samples were flushed with saline solution to remove adherent intestinal contents and then fixed in 10% neutral buffered formaline solution for 24 h. Afterwards taken from formaline and dehydrated in graded ethanol solutions, cleared with xylol and embedded in paraffin, respectively. Then sectioned at the thickness of 6 um with microtome. Mounted sections were stained with Mallory’s modified triple staining technique (Culling et al. 1985; Xu et al. 2003). Villus height was measured from the top of the villus to the crypt mouth and crypt depth was defined as the distance between basements of the crypt-to-crypt mouth. Histological sections were examined under the light microscope.

### Volatile fatty acid (VFA) analysis of cecum contents

After slaughtering, approximately 2 g of cecal digesta was collected and immediately frozen at − 18°C for further analysis. For VFA analysis, caecum samples were thawed at 4°C, diluted, homogenized and then centrifuged at 4000 g for 15 min. The supernatant (1 ml) was taken into an Eppendorf tube and precipitated in an ice bath (for 30 min) after mixing with a metaphosphoric acid solution (25%, 2 ml). The precipitate was removed using centrifugation (11,000g for 10 min) and supernatants were taken for VFA analysis using gas chromatograph (Shimadzu GC‐2010, Shimadzu) coupled with TRB‐FFAP column (Teknokroma, 30 m × 0.53 mm internal diameter) and flame ionization detector (Zhang et al. 2003). The temperature of injector‐port and detector was set at 230°C and 250°C, respectively. Samples were initially held at 120°C for 4 min, then the temperature was increased to 160°C at 4°C/min for 4 more minutes. The volume of injection used was 1 μl and helium was used as the carrier gas (Yalçın et al. 2023).

### Statistical analysis

The statistical analysis was conducted using SPSS 23.0 (SPSS Inc., Chicago, IL, USA). The normality of the data was evaluated using the Kolmogorov–Smirnov test. The means of the data were subjected to one-way ANOVA and significant means were separated using the Tukey test. Polynomial contrasts were also used to determine the linear and quadratic effects of amaranth incorporation into broiler diets. Moreover, the data were analyzed as a 2 × 2 factorial arrangement (excluding the control group), with the amaranth level, heat treatment, and their interactions as the main effects. The level of significance was set at *P* ≤ 0.05.

## Results

The chemical composition of the raw and heat-treated amaranth grain (*A. spinosus*) is presented in Table 2, indicating that there were no major differences between the two types.

**Table 2:** Chemical characteristics (as received) of raw and heat-treated amaranth (*A. spinosus*)

Item	Raw	Heat-treated
Dry matter, %	92.24	91.77
Crude protein, %	15.20	15.10
Ether extract, %	5.35	5.75
Crude ash, %	2.23	2.26
Crude fiber, %	4.10	4.03
ADF, %	7.04	6.65
NDF, %	9.40	9.71

### Performance traits of broiler chickens

The study’s findings on performance traits are summarized in Table 3. Throughout the study, all the birds exhibited good health, and no deaths occurred in either the control or amaranth-incorporated groups. Up to day 24 of the trial (starter and grower periods), the FI of all amaranth-fed groups was comparable to that of the control group (*P* = 0.061, 0.284). However, during the finishing period (25-39d), broiler chickens fed diets with 20% amaranth (either raw or heat-treated) consumed significantly more feed than the control group (*P* = 0.002). This trend was also observed in the total FI (0-39d), with significant differences (*P* = 0.002) between the control group and the 20% amaranth groups (either raw or heat-treated). Polynomial contrast data also showed that the inclusion of amaranth in broiler diets, both raw and heat-treated, linearly increased the FI of birds (0-39d), particularly during the finishing phase (*P* = 0.02, 0.004, respectively). When the main effect of amaranth inclusion was compared, feed consumption increased significantly as the amaranth concentration in the diet was raised from 10 to 20%, and this difference persisted for most of the experiment (*P* = 0.003). Moreover, heat treatment of *A. spinosus* did not alter any of the performance indices of birds (FI, BWG, FCR) when compared to birds fed raw amaranth (*P* > 0.05).

**Table 3:** Effects of raw or heat-treated amaranth (*A. spinosus*) incorporation into broiler diets on FI, BWG and FCR

Treatment^1^	FI, g/bird				BWG, g/bird				FCR, g/g			
	Days											
	0–10	11–24	25–39	0–39	0–10	11–24	25–39	0–39	0–10	11–24	25–39	0–39
Control	309.44	1211.2	2931.1^b^	4451.7^b^	269.17^a^	815.04	1865.2	2949.4	1.15^b^	1.49	1.58^b^	1.51^c^
RW10	305.25	1214.8	3094.6^ab^	4614.7^ab^	249.55^ab^	806.04	1893.3	2949.0	1.23^ab^	1.51	1.64^b^	1.57^b^
RW20	311.75	1265.0	3201.6^a^	4778.4^a^	237.58^b^	796.29	1822.0	2855.9	1.32^a^	1.60	1.76^a^	1.67^a^
HT10	306.79	1233.0	3038.7^ab^	4578.4^ab^	246.73^ab^	785.62	1877.0	2909.3	1.24^ab^	1.57	1.62^b^	1.57^b^
HT20	323.96	1275.0	3189.0^a^	4787.9^a^	250.60^ab^	827.62	1789.0	2867.2	1.29^a^	1.54	1.78^a^	1.67^a^
SEM^2^	2.28	11.47	26.68	34.28	3.27	10.73	19.25	19.62	0.02	0.01	0.02	0.01
*P*-value	0.061	0.284	0.002	0.002	0.029	0.790	0.432	0.422	0.006	0.138	< 0.001	< 0.001
*Polynomial contrasts*												
Control *vs* RW												
* P*-Linear	0.709	0.159	0.001	0.002	0.009	0.650	0.540	0.186	0.004	0.048	< 0.001	< 0.001
* P*-Quadratic	0.326	0.470	0.626	0.996	0.682	0.992	0.417	0.441	0.842	0.431	0.398	0.116
Control *vs* HT												
* P*-Linear	0.136	0.116	0.003	0.004	0.006	0.983	0.345	0.227	< 0.001	0.137	< 0.001	< 0.001
* P*-Quadratic	0.045	0.419	0.180	0.147	0.189	0.179	0.213	0.707	0.637	0.279	0.001	0.001
Main effects^3^												
Amaranth level												
10%	306.02	1223.9	3066.7	4596.5	248.14	795.83	1885.2	2929.1	1.24	1.54	1.63	1.57
20%	317.85	1270.0	3195.3	4783.1	244.09	811.96	1805.5	2861.1	1.31	1.57	1.77	1.67
Heat treatment (HT)												
No	308.50	1239.9	3148.1	4696.5	243.57	801.17	1857.7	2902.4	1.27	1.55	1.70	1.62
Yes	315.38	1254.0	3113.8	4683.1	248.66	806.62	1833.0	2888.2	1.27	1.56	1.70	1.62
* P*-value												
Level	0.020	0.079	0.009	0.003	0.543	0.517	0.058	0.110	0.036	0.344	< 0.001	< 0.001
HT	0.156	0.579	0.450	0.814	0.446	0.825	0.540	0.730	0.882	0.917	0.899	0.853
Level × HT	0.266	0.872	0.632	0.688	0.241	0.302	0.835	0.123	0.490	0.062	0.366	0.646

FI, feed intake; BWG, body weight gain; FCR, feed conversion ratio

^1^Control, non-supplemented diet; RW_10_ and RW_20_, raw amaranth incorporated groups at levels 10 and 20% of the diet, respectively; HT_10_ and HT_20_*,* heat-treated amaranth-incorporated groups at levels 10 and 20% of the diet, respectively

^2^Standard error of mean

^3^Data were analyzed as a 2 × 2 factorial arrangement, excluding control group

Means in column not sharing a common superscript are significantly different (*P* < *0.05*)

The findings on the influence of amaranth inclusion on BWG showed that during the starter phase, BWG was significantly lower in the RW_20_ group (*P* = 0.029) than in the control group. During the same period, the data also revealed a significant linear decrease in BWG as amaranth levels increased, whether raw or heat-treated (*P* = 0.009 and 0.006, respectively). No significant differences in BWG were detected in subsequent stages or over the entire period (0-39d) between the control or amaranth-incorporated groups (either raw or heat-treated) (*P* > 0.05). Increasing the level of amaranth in broiler diets from 10 to 20% had no significant effect on BWG (*P* > 0.05).

The results for feed efficiency also varied considerably, with birds fed 20% amaranth (raw or heat-treated) having a poorer FCR than control birds during the starter and finishing periods (*P* = 0.006 and *P* < 0.001, respectively). For the entire trial (0-39d), birds fed control diets had better FCR than those in the amaranth-incorporated groups (*P* < 0.001). In addition, a significant linear trend in FCR degradation was observed with the introduction of amaranth to the broiler diets, both raw and heat-treated (*P* < 0.001). Furthermore, for both the starter (*P* = 0.036) and finisher (*P* < 0.001) periods as well as for the entire trial (*P* < 0.001), an increase in amaranth level from 10 to 20% led to inferior FCR values.

### Carcass yield and relative organ weights

Table 4 presents the data on key carcass parameters investigated in the study. The carcass yields from all the amaranth-incorporated groups were comparable to those of the control group fed a maize-soybean diet (*P* > 0.05). Additionally, with increasing amounts of amaranth seeds (raw or heat-treated) introduced into broiler diets, the data showed a linear increase in carcass yield (*P* = 0.050 and 0.013, respectively).

**Table 4:** Effects of raw or heat-treated amaranth (*A. spinosus*) incorporation into broiler diets on carcass yield, relative weights of some organs and abdominal fat

	Carcass Traits						
Treatment^1^	%						
	Carcass yield	^Liver^	^Heart^	^Spleen^	^Bursa F^	^Gizzard^	^Abd. Fat^
**Control**	72.91	1.93	0.50	0.11	0.20	1.40^c^	0.77
**RW**_**10**_	73.75	1.92	0.49	0.09	0.21	1.51^bc^	0.76
**RW**_**20**_	73.92	1.94	0.50	0.10	0.19	1.73^a^	0.63
**HT**_**10**_	73.83	1.91	0.49	0.09	0.20	1.58^b^	0.62
**HT**_**20**_	74.10	1.91	0.51	0.11	0.20	1.65^ab^	0.61
SEM^2^	0.15	0.17	0.01	0.003	0.004	0.02	0.03
*P*-value	0.119	0.981	0.852	0.472	0.686	< 0.001	0.127
*Polynomial contrasts*							
Control *vs* RW							
* P*-Linear	0.050	0.926	0.884	0.265	0.267	< 0.001	0.135
* P*-Quadratic	0.436	0.855	0.622	0.294	0.351	0.247	0.403
Control *vs* HT							
* P*-Linear	0.013	0.628	0.660	0.615	0.748	< 0.001	0.032
* P*-Quadratic	0.962	0.935	0.394	0.206	0.921	0.858	0.636
Main effects^3^							
Amaranth level							
10%	73.79	1.92	0.49	0.09	0.20	1.55	0.69
20%	74.01	1.92	0.50	0.10	0.20	1.69	0.62
Heat treatment (HT)							
No	73.84	1.93	0.49	0.10	0.20	1.62	0.70
Yes	73.97	1.91	0.50	0.10	0.20	1.62	0.62
* P*-value							
Level	0.486	0.897	0.305	0.248	0.341	< 0.001	0.240
HT	0.688	0.590	0.670	0.571	0.813	0.888	0.157
Level × HT	0.861	0.859	0.750	0.487	0.329	0.038	0.282

^1^Control, non-supplemented diet; RW_10_ and RW_20_, raw amaranth incorporated groups at levels 10 and 20% of the diet, respectively; HT_10_ and HT_20_, heat-treated amaranth-incorporated groups at levels 10 and 20% of the diet, respectively

^2^Standard error of mean

^3^Data were analyzed as a 2 × 2 factorial arrangement, excluding control group

Means in column not sharing a common superscript are significantly different (P < 0.05)

There were no significant differences (*P* > 0.05) among the control and amaranth-incorporated groups regarding relative organ weights (liver, heart, spleen, bursa of Fabricius) monitored on day 39. The gizzard weight was the lone exception, exhibiting a linear increase in response to the addition of amaranth, both raw and heat-treated (*P* < 0.001). Increasing dietary amaranth levels from 10 to 20% had no significant influence on carcass yield or organ weights (*P* > 0.05), except for the gizzard, where it had an enhancing effect (*P* < 0.001). The data also indicated a significant interaction effect of level × heat treatment on gizzard weight (*P* = 0.038). Furthermore, heat-treated amaranth was found to reduce abdominal fat in a linear pattern (*P* = 0.032).

### Meat pH and birds’ antioxidant status

Changes in the meat pH and antioxidant status of birds fed amaranth and control diets are summarized in Table 5. No adverse effects of amaranth incorporation were observed on meat pH and antioxidant capacity, or blood serum antioxidant status when compared to the control group. The addition of raw amaranth to broiler diets resulted in a linear increase in the pH of the meat (*P* = 0.031). A similar trend was observed in the heat-treated group, but the difference was not statistically significant (*P* = 0.068). The results showed that incorporating amaranth into broiler diets at high levels (20%) negatively affected the meat antioxidant status (TAS) (*P* < 0.001), however, it did not affect the overall balance between the oxidants and antioxidants in the meat (OSI). Additionally, raw amaranth inclusion linearly decreased meat oxidation status (TOS) (*P* = 0.016) and overall oxidative stress index (OSI) (*P* < 0.001). Heat treatment and amaranth levels had no significant effect on MPO, CAT, and DPPH levels in the meat (*P* > 0.05). Furthermore, a linear decrease in blood serum TAS levels was observed when amaranth (raw or heat-treated) was incorporated into the broiler diets (*P* = 0.009, 0.002, respectively). Increasing amaranth to 20% of the diet decreased blood TAS (*P* < 0.001) and increased OSI (*P* = 0.013) when compared to those fed 10% amaranth. The data also indicated a significant interaction effect of level × heat treatment on the blood OSI (*P* = 0.05).

**Table 5:** Effects of raw or heat-treated amaranth (*A. spinosus*) incorporation into broiler diets on meat pH, meat antioxidant parameters and blood serum antioxidant parameters

Treatment^1^	pH	Meat							Blood Serum		
			TAS, mmol/L	TOS, µmol/L	OSI	MPO, U/L	CAT, U/L	DPPH, %	TAS, mmol/L	TOS, µmol/L	OSI
**Control**	6.75		0.47^c^	3.54	0.78^a^	153.36	188.09	0.34	2.02^ab^	5.13	0.26^ab^
**RW**_**10**_	6.90		0.66^a^	3.60	0.55^b^	152.18	215.82	0.46	1.93^ab^	6.06	0.32^ab^
**RW**_**20**_	6.94		0.53^bc^	2.90	0.56^b^	165.83	166.75	0.44	1.77^bc^	6.03	0.34^ab^
**HT**_**10**_	6.89		0.62^ab^	3.43	0.57^ab^	139.58	234.08	0.45	2.04^a^	4.75	0.23^b^
**HT**_**20**_	6.90		0.50^bc^	3.38	0.72^ab^	142.00	224.08	0.39	1.63^c^	6.77	0.42^a^
SEM^2^	0.03		0.02	0.10	0.03	4.66	9.58	0.02	0.03	0.32	0.02
*P*-value	0.191		< 0.001	0.145	0.008	0.393	0.141	0.122	< 0.001	0.276	0.020
*Polynomial contrasts*											
Control *vs* RW											
* P*-Linear	0.031		0.264	0.016	< 0.001	0.451	0.411	0.082	0.009	0.360	0.165
* P*-Quadratic	0.480		< 0.001	0.101	0.009	0.615	0.114	0.152	0.682	0.572	0.735
Control *vs* HT											
* P*-Linear	0.068		0.108	0.605	0.241	0.339	0.179	0.143	0.002	0.254	0.065
* P*-Quadratic	0.740		0.004	0.990	0.056	0.668	0.497	0.057	0.001	0.090	0.010
Main effects^3^											
Amaranth level											
10%	6.89		0.64	3.51	0.56	145.88	224.95	0.46	1.98	5.41	0.28
20%	6.92		0.52	3.14	0.64	153.92	195.42	0.41	1.70	6.40	0.38
Heat treatment (HT)											
No	6.92		0.60	3.25	0.55	159.01	191.28	0.45	1.85	6.05	0.33
Yes	6.89		0.56	3.41	0.65	140.79	229.08	0.42	1.83	5.76	0.32
* P*-value											
Level	0.614		< 0.001	0.094	0.145	0.419	0.170	0.260	< 0.001	0.163	0.013
HT	0.648		0.271	0.485	0.103	0.071	0.081	0.389	0.770	0.682	0.907
Level × HT	0.767		0.670	0.137	0.211	0.571	0.361	0.544	0.055	0.152	0.050

TAS, total antioxidant status; TOS, total oxidant status; OSI, oxidative stress index; MPO, myeloperoxidase; CAT, catalase; DPPH, 2,2-diphenyl-1-picrylhydrazyl

^1^Control, non-supplemented diet; RW_10_ and RW_20_, raw amaranth incorporated groups at levels 10 and 20% of the diet, respectively; HT_10_ and HT_20_, heat-treated amaranth-incorporated groups at levels 10 and 20% of the diet, respectively

^2^Standard error of mean

^3^Data were analyzed as a 2 × 2 factorial arrangement, excluding control group

Means in column not sharing a common superscript are significantly different (P < 0.05)

### Blood biochemical and hematological results

The levels of TP, ALB, and CHL in the blood serum were similar between the amaranth-fed groups and the control group (Table 6). A linear decrease in the serum TG concentration was observed when amaranth (raw or heat-treated) was incorporated into broiler diets (*P* = 0.014, 0.005, respectively). The HT_20_ group presented significantly lower serum TG levels than the control group (*P* = 0.001). Data also showed lower serum TG concentration for 20% amaranth level than 10% (*P* < 0.001). Furthermore, the addition of heat-treated amaranth to broiler diets resulted in a linear increase in the serum IgG levels (*P* = 0.001). The main effects exhibited a significant positive effect of heat treatment on the serum IgG concentration (*P* = 0.012).

**Table 6:** Effects of raw or heat-treated amaranth (*A. spinosus*) incorporation into broiler diets on blood serum biochemical parameters

Treatment^1^	TP, g/dl	ALB, g/dl	CHL, mg/dl	TGs, mg/dl	IgG, mg/dl
**Control**	3.06	1.36	135.49	48.82^ab^	543.67^ab^
**RW**_**10**_	3.10	1.40	127.03	47.87^ab^	542.58^b^
**RW**_**20**_	3.11	1.46	130.49	38.28^bc^	548.08^ab^
**HT**_**10**_	3.07	1.42	129.37	49.30^a^	551.17^ab^
**HT**_**20**_	3.076	1.43	130.53	35.84^c^	553.92^a^
SEM^2^	0.04	0.02	1.73	1.38	1.26
*P*-value	0.994	0.477	0.651	0.001	0.015
*Polynomial contrasts*					
Control *vs* RW					
* P*-Linear	0.704	0.079	0.345	0.014	0.279
* P*-Quadratic	0.856	0.742	0.197	0.226	0.350
Control *vs* HT					
* P*-Linear	0.913	0.196	0.317	0.005	0.001
* P*-Quadratic	0.953	0.858	0.650	0.002	0.946
Main effects^3^					
Amaranth level					
10%	3.08	1.41	128.20	48.58	546.88
20%	3.09	1.44	130.51	37.06	551.00
Heat treatment (HT)					
No	3.11	1.43	128.76	43.08	545.33
Yes	3.07	1.42	129.95	42.57	552.54
* P*-value					
Level	0.937	0.376	0.561	< 0.001	0.141
HT	0.714	0.872	0.765	0.856	0.012
Level × HT	0.972	0.480	0.773	0.488	0.620

TP, total protein; ALB, albumin; CHL, cholesterol; TGS, triglycerides; IgG, immunoglobulin G

^1^Control, non-supplemented diet; RW_10_ and RW_20_, raw amaranth incorporated groups at levels 10 and 20% of the diet, respectively; HT_10_ and HT_20_, heat-treated amaranth-incorporated groups at levels 10 and 20% of the diet, respectively

^2^Standard error of mean

^3^Data were analyzed as a 2 × 2 factorial arrangement, excluding control group

Means in column not sharing a common superscript are significantly different (P < 0.05)

Amaranth incorporation into broiler diets had no significant effect (*P* > 0.05) on blood RBC, HTC, Hb, WBC, or PLT values (Table 7). Heat-treated amaranth significantly increased the MCH values (*P* = 0.020). Moreover, MCHC linearly decreased when raw amaranth was used (*P* = 0.022). The raw and heat-treated amaranth groups presented a linear increase in MCV and RDW (*P* < 0.05). Furthermore, heat treatment significantly increased RDW (*P* = 0.007), while a significant interaction effect of level × heat treatment was noted for MCV (*P* = 0.007).

**Table 7:** Effects of raw or heat-treated amaranth (*A. spinosus*) incorporation into broiler diets on some blood hematological parameters

Treatment^1^	RBCs, 10^6^/µl	HTC, %	MCV, fl	RDW, %	Hb, g/dl	MCH, Pg	MCHC, %	WBC, 10^3^/µl	PLT, 10^3^/µl
**Control**	2.53	33.08	130.68^c^	10.38^c^	11.47	45.06^ab^	34.50	158.38	32.33
**RW**_**10**_	2.50	33.55	133.47^bc^	10.55^bc^	11.64	44.58^b^	33.43	153.82	28.17
**RW**_**20**_	2.34	32.92	137.49^ab^	11.48^ab^	11.12	45.05^ab^	33.17	151.87	27.17
**HT**_**10**_	2.33	31.79	139.31^a^	11.84^a^	10.97	46.38^a^	33.59	157.01	33.08
**HT**_**20**_	2.46	33.28	136.41^ab^	11.68^ab^	11.32	45.55^ab^	33.76	165.60	30.67
SEM^2^	0.04	0.45	0.65	0.16	0.13	0.19	0.19	2.73	1.33
*P*-value	0.286	0.784	< 0.001	0.004	0.519	0.024	0.237	0.564	0.573
*Polynomial contrasts*									
Control *vs* RW									
* P*-Linear	0.145	0.918	< 0.001	0.011	0.443	0.988	0.022	0.476	0.231
* P*-Quadratic	0.566	0.679	0.604	0.304	0.377	0.314	0.403	0.868	0.669
Control *vs* HT									
* P*-Linear	0.145	0.836	< 0.001	0.005	0.439	0.135	0.156	0.484	0.813
* P*-Quadratic	0.059	0.182	0.013	0.288	0.238	0.064	0.526	0.348	0.631
Main effects^3^									
Amaranth level									
10%	2.42	32.67	136.39	11.20	11.30	45.48	33.51	155.42	30.63
20%	2.40	33.10	136.95	11.58	11.22	45.30	33.46	158.73	28.92
Heat treatment (HT)									
No	2.42	33.23	135.48	11.02	11.38	44.81	33.30	152.84	27.67
Yes	2.39	32.54	137.86	11.78	11.14	45.96	33.68	161.31	31.88
* P*-value									
Level	0.843	0.701	0.649	0.228	0.780	0.664	0.916	0.610	0.546
HT	0.785	0.535	0.059	0.020	0.449	0.007	0.387	0.196	0.141
Level × HT	0.108	0.344	0.007	0.085	0.167	0.115	0.627	0.419	0.802

RBCs, red blood cells; HTC, hematocrit; Hb, hemoglobin; WBC, white blood cells; PLT, platelets; MCH, mean corpuscular hemoglobin; MCHC, mean corpuscular hemoglobin concentration; MCV, mean corpuscular volume; RDW, red cell distribution width

^1^Control, non-supplemented diet; RW_10_ and RW_20_, raw amaranth incorporated groups at levels 10 and 20% of the diet, respectively; HT_10_ and HT_20_, heat-treated amaranth-incorporated groups at levels 10 and 20% of the diet, respectively

^2^Standard error of mean

^3^Data were analyzed as a 2 × 2 factorial arrangement, excluding control group

Means in column not sharing a common superscript are significantly different (P < 0.05)

### Intestinal histomorphology

**Fig. 1:**
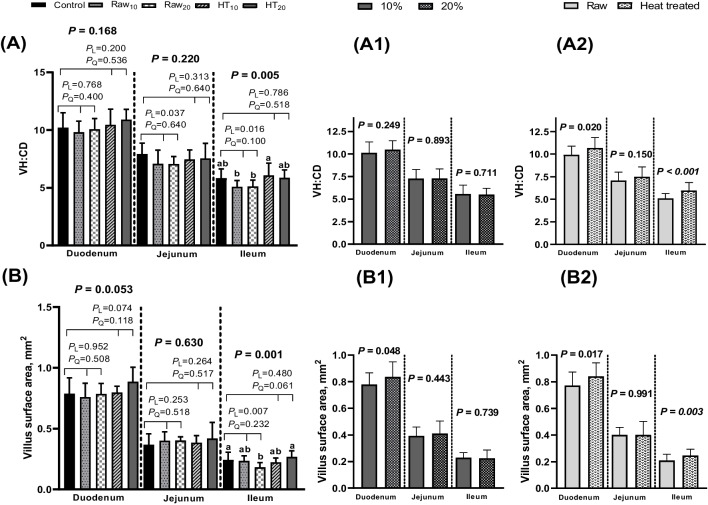
Effects of raw or heat-treated amaranth (*A. spinosus*) incorporation into broiler diets on VH:CD ratio **(A)** and villus surface area **(B)**. A1 and B1, different amaranth levels (10 and 20%); A2 and B2, raw versus heat-treated amaranth

The effects of amaranth incorporation into broiler diets on the histomorphological features of different intestinal sections are shown in Table 8 and Fig. 1. The histomorphological changes (VH, VW, CD, VH:CD, and villus area) observed in the small intestine segments of the group fed amaranth were comparable to those in the control group. Furthermore, the VH of tissue sections from the duodenum increased linearly in the heat-treated amaranth groups (*P* = 0.034). VH in the duodenum increased in response to both the amaranth level and heat treatment (*P* = 0.024 and 0.010, respectively). Raw amaranth linearly decreased the VW in the ileum (*P* = 0.005). In contrast to the negative effect of the amaranth level (*P* = 0.035), heat treatment positively influenced the VW in the ileum (*P* = 0.006). A significant interaction effect of level × heat treatment was noted for VW in the ileum (*P* < 0.001). Furthermore, a shallower ileal CD was observed in the heat treatment group than in the untreated group (*P* = 0.006).

**Table 8:** Effects of raw or heat-treated amaranth (*A. spinosus*) incorporation into broiler diets on small intestine histomorphological features

Treatment^1^	Histomorphological Characteristics, µm								
	Duodenum			Jejunum			Ileum		
	VH	VW	CD	VH	VW	CD	VH	VW	CD
**Control**	1553.46^ab^	160.66	158.34	952.74	122.58	126.12	641.77	121.18^a^	114.80^ab^
**RW**_**10**_	1495.70^b^	161.15	158.49	968.52	131.94	144.63	600.78	123.93^a^	123.00^a^
**RW**_**20**_	1557.75^ab^	159.99	158.91	958.66	133.41	140.49	609.83	94.64^b^	123.76^a^
**HT**_**10**_	1570.22^ab^	161.87	157.71	961.39	126.86	134.80	600.43	119.01^a^	104.95^b^
**HT**_**20**_	1668.46^a^	168.67	159.29	987.89	132.79	139.52	665.66	127.43^a^	119.29^ab^
SEM^2^	16.49	1.94	1.88	18.28	2.10	2.74	10.79	2.96	1.97
*P*-value	0.016	0.629	0.999	0.981	0.424	0.257	0.215	0.002	0.010
*Polynomial contrasts*									
Control *vs* RW									
* P*-Linear	0.936	0.914	0.919	0.901	0.093	0.051	0.423	0.005	0.135
* P*-Quadratic	0.198	0.876	0.978	0.755	0.472	0.074	0.451	0.044	0.457
Control *vs* HT									
* P*-Linear	0.034	0.339	0.935	0.655	0.218	0.165	0.839	0.652	0.915
* P*-Quadratic	0.089	0.453	0.830	0.776	0.625	0.874	0.050	0.425	0.018
Main effects^3^									
Amaranth level									
10%	1532.96	161.51	158.10	964.95	129.40	139.72	600.60	121.47	113.97
20%	1613.11	164.33	159.10	973.27	133.10	140.01	637.74	111.04	121.53
Heat treatment (HT)									
No	1526.73	160.57	158.70	963.59	132.67	142.56	605.30	109.29	123.38
Yes	1619.34	165.27	158.50	974.64	129.82	137.16	633.04	123.22	112.12
* P*-value									
Level	0.024	0.481	0.816	0.848	0.399	0.963	0.092	0.035	0.060
HT	0.010	0.242	0.964	0.799	0.515	0.389	0.205	0.006	0.006
Level × HT	0.599	0.321	0.892	0.676	0.610	0.480	0.199	< 0.001	0.089

VH, villus height; VW, villus width; CD, crypt depth

^1^Control, non-supplemented diet; RW_10_ and RW_20_, raw amaranth incorporated groups at levels 10 and 20% of the diet, respectively; HT_10_ and HT_20_, heat-treated amaranth-incorporated groups at levels 10 and 20% of the diet, respectively

^2^Standard error of mean

^3^Data were analyzed as a 2 × 2 factorial arrangement, excluding control group

Means in column not sharing a common superscript are significantly different (P < 0.05)

The inclusion of raw amaranth in broiler diets linearly decreased the VH:CD ratio in tissue sections from the jejunum and ileum (*P* = 0.037 and 0.016, respectively) (Fig. 1, A). Heat treatment significantly improved the VH:CD ratio in the duodenum and ileum (*P* = 0.020 and *P* < 0.001, respectively) when compared to the untreated group (Fig. 1, A2). The data also showed a linear decrease in the villus area in the ileum with increasing levels of raw amaranth in birds’ diets (*P* = 0.007) (Fig. 1, B). Increasing amaranth to 20% of the diet increased the villus area in the duodenum (*P* = 0.048) compared to the group fed 10% amaranth (Fig. 1, B1). Compared to those in the untreated group, the villus area in the duodenum and ileum was greater in the heat-treated amaranth group (*P* = 0.017 and 0.003, respectively) (Fig. 1, B2). A significant interaction effect of level × heat treatment was noted for the villus area in the ileum (*P* < 0.001).

### Cecal VFA profile

Changes in the cecal VFA profiles of the birds are summarized in Table 9. There were no significant differences (*P* > 0.05) among the control and amaranth-incorporated groups regarding acetic, propionic or butyric acid percentages. The inclusion of raw amaranth in broiler diets linearly decreased (*P* = 0.005) the isobutyric percentage; lower isobutyric acid was present in RW_20_ broilers than in the control (*P* = 0.030). Compared to the control, the addition of amaranth (raw or heat-treated) affected the cecal isovaleric percentage in a positive (*P* = 0.001) or negative (*P* = 0.028) quadratic pattern, respectively. The branched-chain fatty acid (**BCFA**) concentration decreased linearly with amaranth inclusion, whether raw or heat treated (*P* = 0.004 and 0.013, respectively). The raw and heat-treated amaranth groups also presented a linear decrease in the content of valeric acid (*P* < 0.001 and *P* = 0.006, respectively). Heat-treated amaranth linearly decreased the total VFA content in the birds’ cecum (*P* = 0.037). Compared to those in the untreated groups, heat treatment of amaranth significantly increased the concentrations of isobutyric acid, isovaleric acid, and consequently BCFA (*P* < 0.05). A significant interaction effect of level × heat treatment was observed for isovaleric and valeric fatty acids (*P* = 0.003 and 0.006, respectively).

**Table 9:** Effects of raw or heat-treated amaranth (*A. spinosus*) incorporation into broiler diets on cecum volatile fatty acid profile

Treatment^1^	% Total VFAs							mmol/L	
	ACE	PRO	IBA	BUT	IVA	VAL	A/P	BCFA	T. VFAs
**Control**	75.33	8.72	1.42^a^	12.27	1.30^a^	0.96^a^	9.51	2.53^a^	89.31
**RW**_**10**_	76.99	7.51	0.96^ab^	13.13	0.62^b^	0.79^b^	10.71	1.20^b^	75.96
**RW**_**20**_	75.97	10.47	0.84^b^	10.97	1.05^ab^	0.70^b^	8.34	1.28^b^	69.03
**HT**_**10**_	75.21	9.30	1.18^ab^	12.00	1.54^a^	0.76^b^	8.56	2.05^ab^	78.06
**HT**_**20**_	76.40	9.48	1.17^ab^	11.06	1.06^ab^	0.82^ab^	8.69	1.39^b^	63.23
SEM^2^	0.43	0.38	0.06	0.46	0.07	0.02	0.35	0.13	3.37
*P*-value	0.674	0.166	0.030	0.573	< 0.001	< 0.001	0.194	0.001	0.140
*Polynomial contrasts*									
Control *vs* RW									
* P*-Linear	0.654	0.187	0.005	0.388	0.157	< 0.001	0.310	0.004	0.099
* P*-Quadratic	0.282	0.072	0.315	0.246	0.001	0.460	0.081	0.055	0.758
Control *vs* HT									
* P*-Linear	0.586	0.481	0.171	0.511	0.569	0.006	0.370	0.013	0.037
* P*-Quadratic	0.499	0.993	0.710	0.654	0.028	0.095	0.729	0.313	0.419
Main effects^3^									
Amaranth level									
10%	76.10	8.40	1.07	12.57	1.08	0.78	9.64	1.63	77.01
20%	76.19	9.98	1.00	11.02	1.05	0.76	8.51	1.33	66.13
Heat treatment (HT)									
No	76.48	8.99	0.90	12.05	0.83	0.75	9.52	1.24	72.49
Yes	75.81	9.39	1.18	11.53	1.30	0.79	8.63	1.72	70.64
* P*-value									
Level	0.923	0.059	0.590	0.128	0.849	0.540	0.151	0.119	0.065
HT	0.446	0.621	0.029	0.608	0.002	0.087	0.249	0.012	0.749
Level × HT	0.213	0.094	0.648	0.544	0.003	0.006	0.110	0.053	0.496

ACE, acetic acid; PRO, propionic acid; IBA, isobutyric; BUT, butyric; IVA, isovaleric; VAL, valeric; A/P, acetate to propionate ratio; BCFA, branched chain fatty acids (isobutyric + isovaleric); T. VFAs, total volatile fatty acids

^1^Control, non-supplemented diet; RW10 and RW20, raw amaranth incorporated groups at levels 10 and 20% of the diet, respectively; HT10 and HT20, heat-treated amaranth-incorporated groups at levels 10 and 20% of the diet, respectively

^2^Standard error of mean

^3^Data were analyzed as a 2 × 2 factorial arrangement, excluding control group

Means in column not sharing a common superscript are significantly different (P < 0.05)

## Discussion

The inclusion of amaranth (*Amaranthus spp.*) in animal diets, whether as grain or leaf meal, has been shown to provide multiple advantages for animal health and performance. Nevertheless, there are various compounds in amaranth that can impair its nutritive value and hinder growth in poultry. While prior studies have focused predominantly on the use of grain-type amaranth, the present study sought to evaluate the potential of utilizing *A. spinosus* (pigweed) in broiler diets either in its raw form or after heat treatment.

The results revealed that, compared to the control diet, adding amaranth to broiler diets negatively affected the overall FCR. When comparing the two levels of amaranth incorporation, the 20% inclusion performed poorly than the 10%. Furthermore, all performance indices (FI, BWG, FCR) of birds fed heat-treated amaranth were similar to those of birds fed raw amaranth in their diets. The deterioration in FCR was most likely caused by greater FI in birds fed amaranth rather than poor growth, given that no significant changes were observed in BWG. The present study assumed the metabolizable energy (ME) of *A. spinosus* to be approximately 3200 kcal/kg, which was derived from previous research (Tillman et al. 1988; Janmohammadi et al. 2022). However, the ME for *A. spinosus* appeared to be overestimated. Thus, the amaranth inclusion levels in the study may not have been high enough to severely impair the birds' growth response, but they may have been insufficient to meet the birds' energy requirements. As a result, the birds may have increased their FI to compensate for the deficiency, leading to a poorer FCR. To address this issue, further digestibility studies are needed to explore species-specific variations in the metabolizable energy and nitrogen efficiency of amaranth for broilers. Additionally, although the inclusion of whole grains did not restrict feed intake in this study, it may have affected nutrient utilization, leading to insufficient energy intake. However, this hypothesis requires further investigation. Previously, Longato et al. (2017) reported a decrease in BWG in broilers-fed diets containing 5% or 10% raw *A. caudatus* grains compared to those on a control diet. Alizadeh-Ghamsari et al. (2021) reported that when broilers were fed amaranth (*A. hybridus*) up to 6% of the diet, there was no effect on FI, but there was a linear decline in BWG and FCR. According to Orczewska-Dudek et al. (2018), feeding broilers with a diet containing 8% raw amaranth led to reduced FI and BWG but did not significantly alter the FCR when compared to a maize-soybean based diet. Other studies have reported that feeding broilers amaranth grains up to 8% of the diet, whether raw or heat-treated, did not affect final body weight, FI, or FCR (Roučková et al. 2004; Pisarikova et al. 2006). Our findings partially support this view, showing that incorporating *A. spinosus* grains, either raw or heat-treated, into broiler diets at levels up to 10% does not depress growth performance. However, our results contrast with the view that up to 20% of raw or autoclaved amaranth can be added to broiler diets without significantly affecting FI, and FCR (Waldroup et al. 1985). Moreover, experiments with laying hens revealed that including up to 20% autoclaved amaranth (*A. hybridus chlorostachys*) in the diet produced better outcomes in terms of FI and FCR compared to raw amaranth (Hosseintabar-Ghasemabad et al. 2022; Janmohammadi et al. 2023). Overall, the discrepancies among studies regarding the maximum inclusion level of amaranth could be attributed to several factors, including amaranth variety, processing methods (concentration of anti-nutrients), and bird species.

The current results indicated that incorporating *A. spinosus* grains into broiler diets resulted in a similar carcass yield to that of control diet. Furthermore, a linear increase in carcass yield was noted with the rising concentration of amaranth in broiler diets, whether raw or heat-treated. Ren et al. (2023) also observed a higher carcass percentage in broilers consuming diets with 10% inclusion level of *A. hypochondriacus* compared to those on control diets. Higher weights of breast meat or drumsticks have been reported with amaranth inclusion in broiler diets compared to the control diet (Orczewska-Dudek et al. 2018; Manyelo et al. 2022). Amaranth has a high nutritional value compared to cereals, especially in terms of protein and essential amino acids (Hosseintabar-Ghasemabad et al. 2024). The increased carcass yield in this study with the inclusion of amaranth could be related to higher FI and, consequently, greater protein intake, potentially leading to better muscle development and larger carcass size; however, this requires further investigation. Research has also shown no significant difference in carcass yield between control groups and broilers fed either raw amaranth (Orczewska-Dudek et al. 2018) or raw and popped amaranth (Pisarikova et al. 2006) at an 8% dietary inclusion level. Contrary to these results, Alizadeh-Ghamsari et al. (2021) reported that the inclusion of 6% amaranth (*A. hybridus*) in a pelleted diet significantly decreased the carcass yield of broilers, while inclusion levels of 2% and 4% did not exhibit any significant differences compared to control. This could be due to the depressed body weight gain in their study, which was attributed to the presence of antinutrients in amaranth.

The current findings regarding the effects of amaranth inclusion in broiler diets on the relative weights of the heart, spleen, bursa of Fabricius (Alizadeh-Ghamsari et al. 2021) and liver (Pisarikova et al. 2006) are consistent with previous research, showing no adverse effects on these organs when amaranth constituted up to 10% of the diet. Conversely, Orczewska-Dudek et al. (2018) noted heavier livers in broilers fed with 8% raw amaranth compared to control, potentially attributed to the presence of antinutrients in unprocessed amaranth. Furthermore, the inclusion of amaranth in broiler diets has also been shown to reduce abdominal fat (Orczewska-Dudek et al. 2018; Alizadeh-Ghamsari et al. 2021), partly in agreement with the present study’s findings of a linear reduction in abdominal fat with the incorporation of heat-treated amaranth. This indicates the potential hypolipidemic effect of amaranth, as supported by decreased blood serum TG levels in the current study. Decreased abdominal fat might also be related to lower energy uptake by the birds. In contrast, Pisarikova et al. (2006) reported no difference in abdominal fat between control, raw, and popped amaranth-fed broilers. Another significant aspect of amaranth inclusion (whether raw or heat-treated) in broiler diets was the notable increase in gizzard weight with increasing amaranth level, particularly raw amaranth (15% versus 5% increase in weight). The gizzard is an important component of avian digestion, and it tends to increase in size to accommodate fiber in the diet. Thus, the increased gizzard weight observed in this study could be related to the dietary fiber content associated with amaranth. Moreover, heat treatment of amaranth grain might have increased both soluble fiber concentration and fiber digestibility of amaranth (Pedersen et al. 1990), as indicated by a significant interaction effect of level × heat treatment. Alizadeh-Ghamsari et al. (2021) reported no effect on gizzard weight when broilers were fed a pelleted diet containing 6% amaranth (*A. hybridus*). In general, the disparities noted in the findings may predominately arise from differences in amaranth species and processing methods, resulting in variations in nutrient composition, including non-nutritive substances, bioactive elements, fiber levels, etc.

Meat pH plays a crucial role in determining its quality, with a decrease in pH potentially impairing several quality attributes, especially the meat’s water-holding capacity. In the present study, a linear increase in breast meat pH was observed with the inclusion of raw amaranth in broiler diets. This observation is consistent with Manyelo et al. (2022), who reported an increase in the pH of both breast and thigh meat with the addition of *A. cruentus* leaf meal in broiler diets. However, Orczewska-Dudek et al. (2018) found no effect on breast muscle pH when broiler diets included 8% amaranth grain. Similarly, Longato et al. (2017) observed no changes in breast meat pH when broilers were fed *A. caudatus* grain at dietary concentrations of 5%, or 10%. Heat treatment is known to reduce the activity of bioactive substances while inactivating antinutrients (Longato et al. 2017). The higher availability of bioactive compounds in raw amaranth, compared to heat-treated amaranth, might explain the observed changes in meat pH.

Meat quality and the body’s antioxidant status are also closely linked. Several studies have indicated that incorporating amaranth into the diet of birds can enhance their antioxidant capacity. Longato et al. (2017) reported a significant decrease in the serum lipid peroxidation level when broiler chickens were fed *A. caudatus* grain up to 10% of their diet. Similarly, Orczewska-Dudek et al. (2018) demonstrated a strong antioxidant effect of amaranth supplementation in broilers fed diets enriched with polyunsaturated fats. Conversely, Janmohammadi et al. (2023) observed no change in blood antioxidant status (MDA, total antioxidant capacity) in laying hens fed raw *A. hybridus chlorostachys* up to 40% of the diet. However, Hosseintabar-Ghasemabad et al. (2022) found that feeding laying hens with processed (autoclaved) *A. hybridus chlorostachys* (up to 40% of the diet) improved their total antioxidant capacity without affecting MDA levels in the blood. The findings of the present study indicated no adverse effects of amaranth incorporation into broiler diets on meat antioxidant capacity, or blood serum antioxidant status compared to the control group. Additionally, raw amaranth appeared to enhance the meat’s antioxidant capacity more effectively than processed amaranth. In terms of blood serum antioxidant status, the data also revealed better outcomes with a 10% inclusion rate than with 20%. Furthermore, a significant interaction effect of level × heat treatment on the blood OSI indicated the effectiveness of heat treatment in improving the antioxidant potential of amaranth at higher inclusion levels. Amaranth seeds contain various phytochemicals, including polyphenols, tocopherols, squalene, and proteins or peptides with antioxidant activity, which contribute to their high antioxidant potential (Tironi et al. 2010). A higher inclusion of amaranth in broiler diets may lead to inefficient digestion, thus, decreasing the potential release of antioxidant compounds in the animal body. The use of exogenous enzymes in combination with heat treatment may be beneficial for improving the digestion process and, subsequently the antioxidant activity of amaranth grains when fed to broilers.

Hematobiochemical analysis of blood serves as a valuable tool for interpreting the impact of dietary interventions on the internal environment of animals. Healthy blood profiles can act as diagnostic indicators, shedding light on how animals respond to exposure to harmful substances. Amaranth supplementation in poultry diets has been associated with a hypocholesterolemic effect in various studies (Longato et al. 2017; Alizadeh-Ghamsari et al. 2021; Hosseintabar-Ghasemabad et al. 2022; Janmohammadi et al. 2023). Interestingly, the current study revealed no discernable effect on cholesterol levels along with protein or albumin concentrations in the blood when amaranth was introduced into broiler diets, whether raw or heat-treated. Similarly, Popiela et al. (2013) found no evidence of a cholesterol-lowering effect or alterations in total protein or albumin levels upon inclusion of extruded amaranth grains in the diets of laying hens. Studies on broiler chickens have also shown that adding amaranth to their diets has negligible effect on cholesterol (Roučková et al. 2004; Orczewska-Dudek et al. 2018), total protein (Roučková et al. 2004; Alizadeh-Ghamsari et al. 2021) or albumin levels (Longato et al. 2017) in the blood. Furthermore, the present study revealed reduced blood triglycerides following amaranth supplementation (either raw or heat-treated). This may suggest a hypolipidemic role of amaranth, with a more pronounced effect observed at higher inclusion levels. While some studies have reported decreased blood triglycerides (Longato et al. 2017; Orczewska-Dudek et al. 2018), others have found no significant changes (Popiela et al. 2013; Alizadeh-Ghamsari et al. 2021; Janmohammadi et al. 2023). Amaranth is rich in bioactive compounds, such as squalene, phytosterols, and tocopherols, which contribute to its hypocholesterolemic and antioxidant properties. The fatty acid composition of the diet may also influence these effects (Janmohammadi et al. 2023). Inconsistent results across studies may stem from variations in amaranth species, supplementation levels, processing methods, diet compositions, and animal types.

This study demonstrated that amaranth feeding did not compromise birds' immunity, as indicated by IgG levels, which notably increased when heat-treated amaranth was included in the broiler diet. Additionally, amaranth incorporation (whether raw or heat-treated) resulted in variations in some red blood cell indices, including MCV, RDW, MCH and MCHC. However, these indices remained within the normal range, indicating no detrimental effects of amaranth feeding on the physiological status of birds. Króliczewska et al. (2008) also reported unchanged hemoglobin levels and hematocrit volume in laying hens fed amaranth up to 10% of the diet. Further research is needed to fully understand the effects of amaranth levels and processing methods on hematological indices in broiler chickens.

The small intestine assumes a key role in feed digestion and nutrient absorption, thereby constituting an indispensable organ in the body. Assessment of the mucosal structure of the small intestine facilitates estimation of gut health, digestive efficacy, and growth potential in animals. This study revealed that incorporation of amaranth into broiler diets, whether raw or heat-treated, had comparable effects on duodenal, jejunal and ileal morphology relative to the control group. Notably, heat-treated amaranth conferred superior benefits than the raw amaranth, enhancing the VH:CD ratio and villus surface area in the duodenal and ileal segments. Conversely, the jejunum exhibits minimal responsiveness to amaranth supplementation, indicating negligible or marginal impact on its absorptive capacity. Moreover, increasing the level of amaranth (20%) positively influenced the villus surface area of the duodenum, potentially attributed to increased villus height. Conversely, a higher amaranth inclusion rate negatively affected the villus width in the ileum compared to 10%, although no significant effect was observed on the surface area of the villi. Research examining the effects of amaranth feeding on intestinal morphology is limited. In one previous study involving broilers, Alizadeh-Ghamsari et al. (2021) reported results similar to those of the present study, suggesting that amaranth (*A. hybridus*) feeding had no adverse impact on the histomorphological characteristics of the small intestine compared to those of the control group. The changes in intestinal morphology observed herein following amaranth supplementation, irrespective of the processing and inclusion level, may be linked to variations in the digestive enzyme activity and modulation of the cecal microbiota. Nevertheless, further extensive research is needed to better comprehend the impact of amaranth feeding on intestinal health.

Acetic, propionic, and butyric acids are the principal byproducts of carbohydrate fermentation, while isobutyric and isovaleric acids have been suggested as indicators of protein degradation (Elling-Staats et al. 2022). The present study revealed no significant changes in the levels of major VFAs (acetic, propionic, or butyric) in broilers fed diets supplemented with amaranth compared to those in the control group. However, feeding heat-treated amaranth resulted in increased branched-chain fatty acids and valerate in the ceca of broilers, which may suggest a negative effect of heat on the protein quality of the grain. Despite these changes, the overall concentration of VFAs remained similar across all groups.

## Conclusions

The substitution of *A. spinosus* for conventional grains in broiler diets revealed that while amaranth had no detrimental impact on body weight gain of the birds, its presence in the diet had a negative influence on feed conversion ratio, owing to greater feed intake. Feeding amaranth to broiler chickens had no negative effect on carcass yield or organ weights, except for the gizzard, which was heavier in the amaranth-fed groups. Furthermore, amaranth feeding reduced abdominal fat content and demonstrated the ability to withstand changes in meat pH while improving meat antioxidant status. The addition of amaranth to broiler diets had no adverse effects on blood biochemical or hematological indices. Although heat treatment of amaranth did not significantly affect the performance of broiler chickens, it improved the beneficial effects of amaranth feeding on the histological features of the duodenum and ileum and strengthened immunity by boosting immunoglobulin G levels. Overall, the findings of this study contribute to the growing body of knowledge on utilizing sustainable feed sources, such as amaranth, for poultry production, ultimately promoting more sustainable and environmentally conscious agricultural practices. More research is needed to determine the impact of various amaranth species on poultry nutrition, including different processing methods and enzyme supplementation.

## Data Availability

The data generated and analyzed during the current study are not publicly available due to privacy reasons. However, upon reasonable request, the data can be made available by contacting Dr. Emre Sunay Gebeş at esgebes@ankara.edu.tr. We are committed to facilitating scientific reproducibility and transparency and will provide access to the data in accordance with applicable privacy and confidentiality regulations.
